# Size Matters: Non-Numerical Magnitude Affects the Spatial Coding of Response

**DOI:** 10.1371/journal.pone.0023553

**Published:** 2011-08-11

**Authors:** Ping Ren, Michael E. R. Nicholls, Yuan-ye Ma, Lin Chen

**Affiliations:** 1 State Key Laboratory of Brain and Cognition, Kunming Institute of Zoology, Chinese Academy of Sciences, Kunming, Yunnan, People's Republic of China; 2 Graduate University of Chinese Academy of Sciences, Beijing, People's Republic of China; 3 School of Psychology, Flinders University, Adelaide, Australia; 4 State Key Laboratory of Brain and Cognition, Institute of Biophysics, Chinese Academy of Sciences, Beijing, People's Republic of China; Monash University, Australia

## Abstract

It is known that small and large numbers facilitate left/right respectively (the SNARC effect). Recently, it has been proposed that numerical magnitude is just one example of a range of quantities, which have a common cognitive/neural representation. To investigate this proposition, response congruency effects were explored for stimuli which differed according to their: (a) numerical size, (b) physical size, (c) luminance, (d) conceptual size and (e) auditory intensity. In a series of experiments, groups of undergraduate participants made two-alternative forced choice discriminations with their left or right hands. There were clear interactions between magnitude and responding hand whereby right hand responses were faster for stimuli with (a) large numbers, (b) large physical size, (c) low luminance, and (d) a reference to large objects. There was no congruency effect for the auditory stimuli. The data demonstrate that the response congruency effect observed for numbers also occurs for a variety of other non-numerical visual quantities. These results support models of general magnitude representation and suggest that the association between magnitude and the left/right sides of space may not be related to culture and/or directional reading habits.

## Introduction

Besides having an obvious arithmetical quality, it is becoming increasing apparent that numbers can also have a spatial quality. The link between numerical magnitude and space is clearly demonstrated by the SNARC (spatial numerical association response code) effect [1], [2]. In a classic demonstration of this effect, Dehaene and colleagues found that left- and right-hand responses are faster to small and large numbers (respectively) for number comparison and parity judgments. To explain this effect, Dehaene proposed the existence of a ‘mental number line’, where small and large numbers are represented on the left and right sides of the line, respectively. Responses to small numbers are therefore faster for the left hand because this hand is located in the left hemispace and there is a natural association between the stimulus representation and response code (and vice versa for large numbers) [1], [2]. The SNARC effect appears to extend beyond numbers and occurs for other ordinal quantities such as letters of the alphabet and calendar events [3], [4].

Despite the reliability of the SNARC effect [5], [6] the mechanisms that underlie it are still a matter of debate. In terms of the effectors, research demonstrates that the SNARC effect is not limited to the hands and also occurs for the feet and fingers within a hand [7]–[9]. In addition, associations between numerical magnitude and space have been observed for tasks that do not require a left/right response – suggesting that numbers can produce a shift of attention independent of response [10].

The direction in which text is habitually read may explain the link between ordinal sequences such as numbers and space. Although reading direction may not specifically affect the perception of numbers, it may have a more generalized effect whereby stimuli are scanned in a direction that is consistent with reading habits. Thus stimulus features and responses on the left may be prioritized in left-to-right readers of English. Zebian [11] investigated the mental number line in cultures that read from left-to-right (English monoliterates) and right-to-left (Arabic monoliterates). For English readers, a typical SNARC effect was observed, which was reversed for readers of Arabic. While the study by Zebian suggests that reading direction plays a role in the SNARC effect, the picture does seem to be more complicated than this. For example, studies using the SNARC paradigm have found evidence for a vertical effect where small and large numbers are located towards the bottom and top of space, respectively [12], [13]. A vertical effect such as this would not be predicted by reading direction.

The link between numerical magnitude and space could also reflect the activation of a common set of neural mechanisms. Patients with right parietal damage and left neglect systematically mis-bisect physical and numerical lines to the left [14] – suggesting a common neurological origin. Further support comes from functional magnetic resonance imaging studies, which demonstrate that the intraparietal sulcus plays an important role in number and space information processing [15], [16]. The role of the intraparietal sulcus extends to magnitudes other than numbers and has been observed for quantities such as size, luminance and time [17]. Transcranial magnetic stimulation over the intraparietal sulcus has also been found to disrupt numerical and other magnitude processes [18]–[20].

An insight into the mechanisms that underlie the association between numbers and space may come from the ATOM (a theory of magnitude) model proposed by Walsh [21]. This model proposes a system of generalized magnitude representations serving diverse quantifiable dimensions. Instead of a SNARC, Walsh proposed a SQARC (spatial quantity association response code). A central tenet of this theory is that the brain represents magnitudes across different dimensions (e.g. space, time and numerosity) using a common abstract magnitude code rather than via distinct specific representations unique to each particular dimension. In support of this model, links have been observed between quantities such as time and size [22] and time and space [23]. In specific relation to left/right representations, researchers have found interactions between task-irrelevant numbers and characteristics such as color, size and luminance [17], [24]. Whether SNARC effect can be extended to a broad range of magnitudes, which have no numerical value, however, remains largely untested.

In present study, we applied the SNARC paradigm to a broad range of non-numerical stimuli. The basic methodology required participants to make a judgment of relative magnitude between a sequentially presented reference and target stimulus, which differed along a dimension such as size or luminance. Responses were made with the hands placed in the left- and right-hemispaces and it was expected that responses would be faster when hand placement was congruent with the mental representation of the stimulus. In the first experiment, participants made judgments related to numerical magnitude and a typical SNARC-type congruency effect was expected. In Experiment 2, participants made judgments of physical size for pairs of disks. If physical size maps onto numerical size, a similar congruency effect should occur. In Experiment 3, participants made luminance judgments between pairs of disks. If ‘light’ and ‘dark’ map onto other quantities such as ‘small’ and ‘large’, a similar congruency effect would be predicted. Experiment 2 and 3 will directly manipulate the quantity of a stimulus by altering its physical appearance. In Experiment 4, we investigated whether concepts of different sizes could produce a similar effect. In this case, participants were shown words referring to objects of different size and made judgments along this dimension. Finally, in Experiment 5, participants listened to tones of different intensity and made judgments of sound volume. If the findings from the previous visual experiments generalize to the auditory modality, a similar congruency effect should be observed.

## Method

### Ethics statement

This study was conducted according to the principles expressed in the Declaration of Helsinki and had approval from the Human Research Ethics Committee of the Institute of Biophysics, Chinese Academy of Sciences. All participants provided written informed consent for the collection of data and subsequent analysis.

### Participants

Undergraduate students (n = 98) were paid to participate in the experiment and were split between the five experiments. The participant characteristics for each experiment were as follows: Experiment 1 (f = 7, m = 9, mean age = 22, range = 20–25), Experiment 2 (f = 10, m = 9, mean age = 23, range = 20–25), Experiment 3 (f = 13, m = 10, mean age = 23, range = 19–26), Experiment 4 (f = 11, m = 8, mean age = 22, range = 18–24), Experiment 5 (f = 10, m = 11, mean age = 21, range = 18–25). All participants had normal hearing, normal or corrected-to-normal vision and were naive to the purpose of these experiments. No participant completed more than one experiment.

### Apparatus

The experiments were conducted in a dimly-lit sound-attenuated room. Participants were seated in a chair facing a screen with their head kept sill in a chin-rest. Stimuli were viewed at a distance of 700 mm. Auditory stimuli were delivered via headphones (Sennheiser HD-215). Visual stimuli were presented on a 19-inch CRT monitor with a resolution of 1024×768 pixels and a refresh rate of 100 Hz. Participants responded by using the computer's keyboard. The experimental procedure was created with Matlab 6.5 using the psychtoolbox 2.54 toolbox.

### Stimuli and Procedure

#### Experiment 1

**Figure 1:**
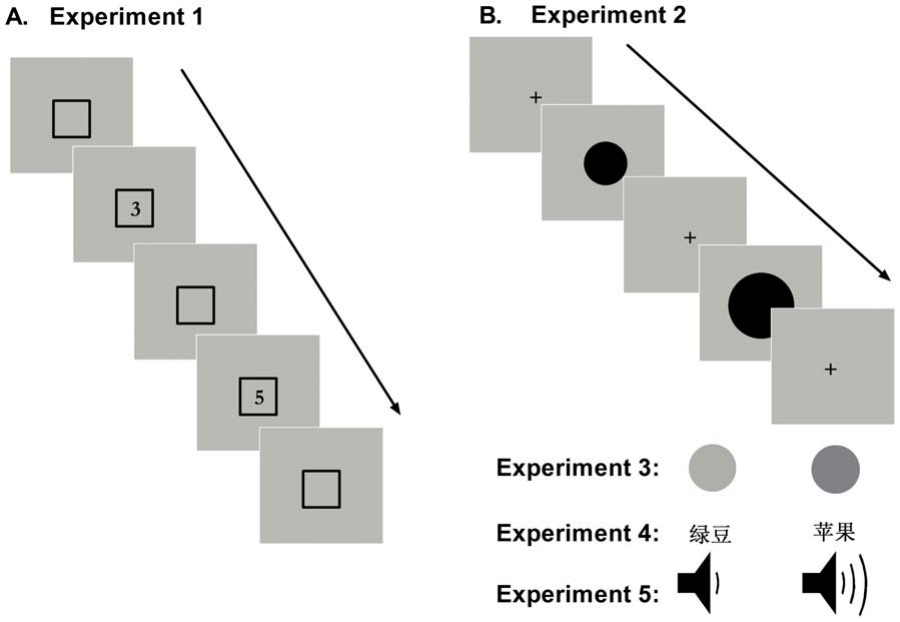
Diagram of the experimental procedure for each task. (a) Experiment 1: numerical magnitude (b) Experiment 2: physical magnitude, (c) Experiment 3: luminance, (d) Experiment 4: conceptual magnitude and (e) Experiment 5: sound intensity. Flow diagrams are not shown for Experiments 3–5 because they were effectively identical to the procedure used for Experiment 2.

Each trial began with the presentation of an unfilled black square (26×26 mm) placed around the centre of the screen. The square remained on through the trial. After 300–800 ms, a digit was presented in the centre of the screen for 300 ms (the *reference* stimulus). The digit was written in ‘Times New Roman’ font and was 18 mm high and 12 mm wide. The digit was presented in black against a gray background. The display was then cleared except for the square for 500 ms. A second digit then appeared in the screen’s centre for 300 ms (the *target* stimulus). Figure 1 illustrates the stimulus sequence. The digits ranged in numerical size between 1 and 9 and were arranged into 10 ascending ( [3], [4], [3], [5], [4], [5], [4], [6], [5], [6], [5], [7], [6], [7], [6], [8], [7], [8] & [7], [9]) and 10 descending pairs ([3], [2], [3], [1], [4], [3], [4], [2], [5], [4], [5], [3], [6], [5], [6], [4], [7], [6] & [7], [5]). Following the presentation of the second digit, participants indicated as quickly as possible whether the second digit was numerically smaller or larger than the first. Responses were made with the index finger of the left and right hands on the ‘S’ and ‘L’ keys of a keyboard, respectively. The keys were covered with a red marker to hide their identity. Participants responded ‘smaller’ and ‘larger’ with their left and right hands, respectively for half of the trials. For the other half of trials, hand of response was reversed. Hand of response was varied between two blocks of 60 trials (3×20 digit combinations) with order controlled between participants. Before the experimental trials, participants were given 10 practice trials to familiarize them with the task.

#### Experiment 2

This experiment was essentially the same as Experiment 1 except for the following points. Each trial began with a fixation cross instead of the square and the fixation cross re-appeared during the 500 ms break between the reference and target presentations. Instead of digits, the stimuli were filled disks of nine different diameters. The diameters were: 35, 52, 70, 87, 104, 121, 138, 156 and 173 mm. The disks were presented in black against a gray background. Like the digits, the disks were arranged into 10 ascending and 10 descending pairs. The manner in which the disk pairs were arranged was the same as the digits (if one thinks of the smallest disk as a ‘1’ and the largest as a ‘9’). Participants indicated whether the second disk was physically smaller or larger. Hand of response was blocked and controlled like Experiment 1.

#### Experiment 3

This experiment used the same disks as described for Experiment 2. In this case, however, the size of the disks was held constant at a diameter of 104 mm. The disks changed in luminance in nine steps from 2.3 cdm^2^ to 22 cdm^2^ Stimulus sequence pairs were created using the procedure for Experiment 2. Participants indicated whether the second stimulus was darker or lighter and hand of response was blocked and controlled.

#### Experiment 4

In this experiment, participants were shown nine words referring to objects of different size. The words were: ‘
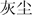
’ (dust), ’
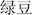
’ (bean), ’
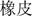
’ (eraser), ’
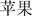
’ (apple), ’
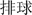
’ (volleyball), ’
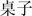
’ (table), ’
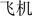
’ (plane), ’
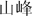
’ (mountain). Participants judged whether the second word referred to an object that was smaller or larger than the first word. Because this task was more difficult, the time interval between reference and target was extended to 1000 ms.

#### Experiment 5

The final experiment used auditory stimuli presented through headphones. Pure sine wave tones (1 KHz) were presented for 300 ms with a 1000 ms interval between them. The tones were presented at nine different intensities ranging from 75 dB to 95 dB (measured using CEM DT-805). To make the task easier, the 9 sound levels were arranged into 5 ascending ([3], [5], [4], [6], [5], [7], [6], [8] & [7], [9]) and descending pairs ([3], [1], [4], [2], [5], [3], [6], [4] & [7], [5]). The experiment was divided into two blocks of 60 trials (6×10 sound combinations) for a total of 120 trials. Participants indicated whether the second tone was louder or quieter than the first tone.

## Results

For all experiments, trials with an incorrect response or with an RT exceeding 3 deviations from the mean RT were excluded. Mixed model analyses of variance (ANOVAs) were used to analyze the RT data with response (left/right) and target (larger/smaller, darker/lighter etc) as within subjects factors and block-order as a between subjects factor. For all experiments, the between subjects factor was not significant and is not reported further. Greenhouse–Geisser corrections were used to correct for unequal variances.

### Experiment 1

**Figure 2:**
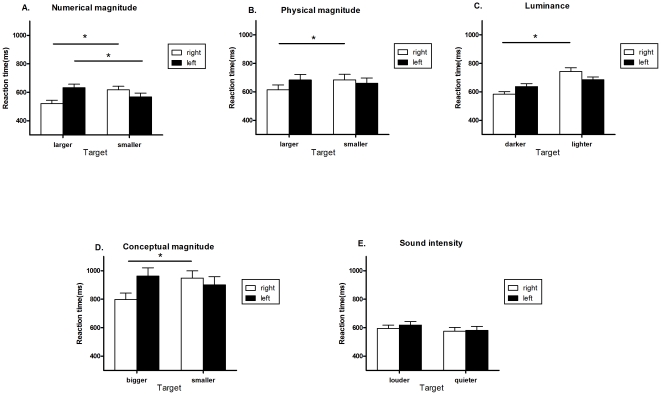
Results of experiments. Graphs showing mean RT data (with SE bars) for the different targets as a function of response (left/right hand) for Experiments 1–5 (Figures 2a–2e). Significant differences for post-hoc tests are indicated (*, p<.05).

The average error rate was 10.7%. There was no sign of a speed/accuracy trade-off. For the RT data, there was a main effect of response whereby responses made by the right hand (Mean: 569 ms, SD: 106.581, RSD: 18.7%) were faster than the left hand (Mean: 600 ms, SD: 105.477, RSD: 17.6%) [F (1, 14)  = 19.648, p<0.001]. There was a trend for responses to be faster to numerically-larger targets (Mean: 576 ms, SD: 110.216, RSD: 19.1%) compared to numerically-smaller targets ((Mean: 593 ms, SD: 103.462, RSD: 17.5%) [F (1, 14)  = 4.202, p = 0.06]. Importantly, Figure 2a shows a significant interaction between response and target [F(1, 14) = 30.679,p<0.001]. Post-hoc t-tests revealed that responses with the right hand were faster to numerically-large (Mean: 521 ms, SD: 92.262, RSD: 17.7%) compared to numerically-small targets (Mean: 617 ms, SD: 100.034, RSD: 16.2%), p<0.001. In contrast, responses with the left hand were faster to numerically-small (Mean: 569 ms, SD: 104.187, RSD: 18.3%) compared to numerically-large targets (Mean: 632 ms, SD: 99.935, RSD: 15.8%), p = 0.029.

### Experiment 2

The average error rate was 2.5%. There was no sign of a speed/accuracy trade-off. For the RT data, responses with the right hand (Mean: 649 ms, SD: 161.539, RSD: 24.9%) were faster than for the left hand (Mean: 671, SD: 164.799, RSD: 24.6%), resulting in a significant main effect of hand [F (1, 17)  = 4.966, p = 0.04]. There was a trend for responses to large targets to be faster (Mean: 648 ms, SD: 162.735, RSD: 25.1%) than to small targets (Mean: 672, SD: 163.532, RSD: 24.3%) [F (1, 17)  = 4.28, p = 0.054]. Figure 2b shows a significant interaction between response and target [F(1, 17) = 4.577,p = 0.047]. Post-hoc t-tests revealed that responses with the right hand were faster to large (Mean: 614 ms, SD: 152.850, RSD: 24.9%) compared to small targets (Mean: 684 ms, SD: 166.276, RSD: 24.3%), p = 0.026. In contrast, there was no significant difference for left hand responses to small (Mean: 659 ms, SD: 164.340, RSD: 24.9%) compared to large targets (Mean: 683 ms, SD: 168.918, RSD: 24.7%), p = 0.218.

### Experiment 3

The average error rate was 6.5%. There was no sign of a speed/accuracy trade-off. For the RT data, there was no effect of response hand (Right hand, Mean: 664 ms, SD: 132.351, RSD: 20%; Left hand, Mean: 661 ms, SD: 99.505, RSD: 15.1%) [F(1, 21) <1]. Responses to dark targets were faster (Mean: 610 ms, SD: 98.158, RSD: 16.1%) than for light targets (Mean: 714 ms, SD: 110.606, RSD: 15.5%) (F (1, 21)  = 65.234, p<0.001). Figure 2c shows a significant interaction between response and target F(1, 21) = 8.722,p = 0.008]. Post-hoc t-tests revealed that responses with the right hand were faster to dark (Mean: 583 ms, 89.675, RSD: 15.4%) compared to light targets (Mean: 744 ms, SD: 119.787, RSD: 16.1%), p<0.001. Within the left hand, there was a trend for faster responses to dark (Mean: 637 ms, SD: 100.936, RSD: 15.8%) compared to light targets (Mean: 685 ms, SD: 94.091, RSD: 13.7%), p = 0.053.

### Experiment 4

The average error rate was 7.2%. There was no sign of a speed/accuracy trade-off. For the RT data, responses with the right hand (Mean: 873 ms, SD: 219.690, RSD: 25.2%) were faster than for the left hand (Mean: 932 ms, SD: 248.393, RSD: 26.7%) [F (1, 17)  = 12.255, p = 0.003]. Responses to big targets (Mean: 879 ms, SD: 238.951, RSD: 27.2%) were faster than to small targets (Mean: 926 ms, SD: 231.502, RSD: 25.0%) [F (1, 17)  = 6.106, p = 0.024]. Figure 2d shows a significant interaction between response and target, [F(1, 17) = 9.04,p = 0.008]. Post-hoc t-tests revealed that responses with the right hand were faster to big (Mean: 798 ms, SD: 196.969, RSD: 24.7%) compared to small targets (Mean: 949 ms, SD: 219.997, RSD: 23.2%), p<0.001. Within the left hand, there was no difference in response times to small (Mean: 902 ms, SD: 246.204, RSD: 27.3%) compared to big targets (Mean: 962 ms, SD: 253.561, RSD: 26.4%), p = 0.197.

### Experiment 5

The average error rate was 5.1%. There was no sign of a speed/accuracy trade-off. For the RT data, There was no effect of response hand (Right hand, Mean: 585 ms, SD: 113.469, RSD: 19.4%; Left hand, Mean: 599 ms, SD: 121.370, RSD: 20.3%) [F (1, 19)  = 1.547, p = 0.229]. Responses to quieter targets (Mean: 578 ms, SD: 121.711, RSD: 21.1%) were faster than for louder targets (Mean; 606 ms, SD: 111.768, RSD: 18.4%) [F (1, 19)  = 7.17, p = 0.015]. There was no interaction between response and target F(1, 19)<1] (see Figure 2e).

## Discussion

The different experiments yielded a number of consistent results. First, there was an effect of hand across most of the experiments whereby right-hand responses were faster than left-hand responses. This asymmetry most likely reflects the superior motor ability of the right hand in the majority of the population [25]. There was also an effect of target. Responses were faster when the target was numerically larger, physically larger, darker, conceptually larger and quieter than the reference. Given that participants had to respond to both types of target and that the targets appeared with equal frequency, this effect cannot be the result of a simple contingency effect. Instead, the effect of target may be related to the temporal contingency between the reference and target stimulus. It is known that dimensions such as number and size not only have spatial qualities [1], but also have temporal properties [26]. For example, Müller and Schwarz [27] found that ascending sequences (e.g 1–2) were associated with faster responses and fewer errors than descending sequences (e.g. 2–1). In the present case, trials where a higher magnitude target followed a lower magnitude reference may have been facilitated because they were aligned with the ascending advantage. While this explanation accounts for the advantage observed for targets that were larger or darker, it does not account for the advantage for quieter targets, which presumably were in a descending order.

Of particular importance to the current hypotheses, most of the experiments showed an interaction between response and target. In Experiment 1, participants made relative judgments of numerical magnitude. Responses to small and large targets were faster with the left and right hands, respectively, and this effect is consistent with the SNARC effect. Previous studies of the SNARC effect have tended to use a standard reference throughout the experiment (i.e. is the target smaller or larger than 5?) [1]. In the current study, we used a dynamic reference, which changed each trial. This technique, which may have encouraged participants to concentrate on relative magnitude across the numerical range, produced a strong SNARC effect.

In Experiment 2, participants made relative judgments of physical size for two disks, which had no numerical value. The ATOM theory proposed by Walsh [21] proposes that number is just one of a number of magnitudes that share a common representation in the brain. In support of this, Pinel [15] found overlapping neural circuits in the parietal cortex for the representation of number, size and luminance for comparison judgments. In addition, research has shown that transcranial magnetic stimulation over the intraparietal sulcus can interfere with judgments related to relative size (e.g. which is the larger object: ‘bikini’ or ‘coat’?) [28]. The results showed a congruency effect where responses to physically small and large targets interacted with response hand. In the case of right hand responses, there was a significant reaction time advantage for large targets. The form of this congruency effect is analogous to the SNARC effect and suggests that physical and numerical size have a common left/right representation.

Experiment 3 explored whether the effects observed for numerical and physical size could be extended to a luminance judgment, which is not associated with size. Although no other studies have explicitly explored this issue, Fias et al. [29] explored whether irrelevant numerical magnitude could affect judgments along dimensions such as orientation, shape or color. They found that numerical magnitude could produce SNARC effects for judgments of orientation, but not for color or shape. They argued that orientation may be processed by parietal circuits, but color and shape were not, which is counter to the claim made by Pinel [17]. The data collected in this experiment showed a clear congruency effect consistent with the hypothesis that the concepts of ‘light’ and ‘dark’ are associated with the left and right sides of space respectively.

Size was manipulated in Experiment 2 by directly changing the physical appearance of the stimulus. Experiment 4 explored whether a similar effect could be induced though conceptual activation of size. The results demonstrated that conceptual activation, by referring to objects of different sizes (e.g ‘table’ or ‘mountain’), produced a congruency effect that was very similar to that observed for Experiment 2. The results therefore demonstrate that the target/response congruency effect is not tied to the immediate physical properties of the stimulus, but relate to a higher-order representation. In this sense, the results are analogous to the SNARC effect itself where congruency effects have been reported for digits as well for enumeration judgments [30]. The results are also relevant to research that has demonstrated spatial-temporal interactions for words referring to past and future events [23], [31] and to research linking numerical knowledge ability and the linguistic comprehension of relative quantifiers [32].

In the final experiment, we examined whether the congruency effects observed in the previous experiments could be extended to the auditory modality. Previous research has demonstrated that the SNARC effect is amodal and that auditory presentations of numbers can produce a SNARC effect [30]. In addition, pitch has been found to have a left-to-right organization, leading to a so-called ‘Spatial, Musical Association of Response Codes’ [21]. The current study explored the more general hypothesis that the ‘magnitude’ of the sound (i.e. the sound intensity) may have a spatial organization. This proposition seems reasonable given that regional cerebral blood flow research implicates fronto-parietal regions during intensity discriminations [33]. Despite this, there was no sign of an interaction between target and response. This suggests that, whereas visual magnitude has a clear left/right mental representation, auditory stimuli do not. It is possible that auditory stimuli have a spatial structure that is less clear than visual stimuli. In support of this, research has found that auditory system has a low spatial sensitivity with respect to visual system [34], [35]. Thus, spatial congruency effects may only occur for auditory stimuli that have a cultural coding of left/right – such as numbers and pitch as it is represented on a musical keyboard.

Throughout the current experiments, participants made explicit judgments of magnitude. In this sense, they are analogous to studies of the SNARC effect where participants make magnitude judgments (i.e. is ‘2’ smaller or larger than ‘5’?) [1]. However, the SNARC effect has also been demonstrated where numerical magnitude is irrelevant to the response using parity judgments (ie. Is ‘2’ odd or even?) [2]. The latter experiments are particularly interesting because they demonstrate that numbers automatically evoke a sense of magnitude – even when it is irrelevant to the task. Although the current study has demonstrated the dimensions such as size and luminance have a spatial dimension, we have not established whether or not this is automatic. Future research could adapt the methodology used in the current study to make the response orthogonal to the dimension of interest. For example, Experiment 2 could be repeated using disks and squares of different sizes. Instead of indicating whether the stimulus was smaller or larger, participants would indicate whether the shape was a circle or a square. If the left/right coding of a square is automatic, there should be a congruency effect between response and size even though size is irrelevant to the task.

In summary, the data demonstrate consistent target/response congruency effects for a variety of magnitudes other than numbers. These results are consistent with the ATOM model [21], which proposes that a variety of different magnitudes are represented along a common cognitive/neural framework. The data are also relevant to the idea of ‘polarity correspondence’ proposed by Proctor and Cho [36]. They demonstrated that stimulus-response mappings could be based on a structural similarity whereby stimulus and response alternatives are mapped along a positive/negative polarity. Such congruency effects in polarity mappings could also explain the associations observed in this study. For example, if left/right, smaller/larger and bright/darker are coded as negative and positive (respectively), a response congruency effect similar to the one observed in the current study would be observed.

The results also have implications for theories emphasizing the contribution of cultural and/or reading habits to the SNARC effect [11]. While it seems plausible that reading habits could affect the spatial representation of numbers, it is more difficult to envisage how reading could affect the left/right representation of qualities such as size and luminance. The results therefore suggest that the left/right spatial representation of magnitude is grounded in a cognitive/neural mechanism that is not culture-specific.
